# Multi-phase simultaneous segmentation of tumor in lung 4D-CT data with context information

**DOI:** 10.1371/journal.pone.0178411

**Published:** 2017-06-16

**Authors:** Zhengwen Shen, Huafeng Wang, Weiwen Xi, Xiaogang Deng, Jin Chen, Yu Zhang

**Affiliations:** 1 School of Biomedical Engineering, Southern Medical University, Guangzhou, Guangdong, China; 2 Guangdong Provincial Key Laboratory of Medical Image Processing, Southern Medical University, Guangzhou, Guangdong, China; 3 Nanfang Hospital, Southern Medical University, Guangzhou, Guangdong, China; Chongqing University, CHINA

## Abstract

Lung 4D computed tomography (4D-CT) plays an important role in high-precision radiotherapy because it characterizes respiratory motion, which is crucial for accurate target definition. However, the manual segmentation of a lung tumor is a heavy workload for doctors because of the large number of lung 4D-CT data slices. Meanwhile, tumor segmentation is still a notoriously challenging problem in computer-aided diagnosis. In this paper, we propose a new method based on an improved graph cut algorithm with context information constraint to find a convenient and robust approach of lung 4D-CT tumor segmentation. We combine all phases of the lung 4D-CT into a global graph, and construct a global energy function accordingly. The sub-graph is first constructed for each phase. A context cost term is enforced to achieve segmentation results in every phase by adding a context constraint between neighboring phases. A global energy function is finally constructed by combining all cost terms. The optimization is achieved by solving a max-flow/min-cut problem, which leads to simultaneous and robust segmentation of the tumor in all the lung 4D-CT phases. The effectiveness of our approach is validated through experiments on 10 different lung 4D-CT cases. The comparison with the graph cut without context constraint, the level set method and the graph cut with star shape prior demonstrates that the proposed method obtains more accurate and robust segmentation results.

## I. Introduction

The accurate radiation target definition for a moving object, such as a lung tumor, in cancer radiation therapy has been drawing much attention for the past decades. An accurate target definition enables the precise delivery of a high-radiation dose to tumor and maintains a low dose to the surrounding organs at risk [1]. The information used for target definition usually comes from images acquired by computed tomography (CT). CT images have an advantage in spatial resolution, but ordinary free-breathing CT scan cannot synchronously characterize the anatomic motion and deformation caused by respiration. Artifacts caused by this motion and deformation are commonly observed in thoracic CT images. These artifacts cause distortion of the target volume and incorrect positional and volumetric information, which make an accurate target definition a challenging problem. Therefore, implementing a precise dose delivery and protecting healthy organs are difficult.

Precise radiation planning and dose have become possible in lung cancer radiotherapy by the use of lung 4D computed tomography (4D-CT). 4D-CT is usually obtained by sorting multiple free-breathing CT segments depending on the respiratory motion recorded by extra instruments and internal anatomical features. The respiratory motion information captured by lung 4D-CT is tremendously valuable for precise target definition. Lung 4D-CT has been routinely used during lung cancer radiotherapy [2, 3, 4]. As regards the current tumor radiotherapy, oncology doctors still manually outline the target slice-by-slice. However, the lung 4D-CT dataset for each patient contains many times of slices more than the 3D-CT data, which makes manual target delineation a heavy workload for doctors. Thus, computer-aided image segmentation techniques, which can guarantee satisfying segmentation results, are eagerly needed to help doctors delineate the tumor.

In recent works, different methods have been proposed to segment lung tumors. Cui et al. [5] presented a prior knowledge enhanced random walk to segment lung tumor from low contrast CT images. The authors used prior knowledge acquired from PET to automatically select foreground seeds, background seeds and the walking range to increase the speed and accuracy of lung tumor segmentation. Based on the “click & grow” algorithm, Gu et al. [6] proposed a single click ensemble approach to delineate lung tumors. An ensemble segmentation was obtained from multiple regions that were grown from different seed points followed by voting. Vivanti et al. [7] proposed a method for the segmentation of lung tumors. Their method consists of four steps: deformation registration, segmentation of lung tumor, leaks detection and tumor boundary regularization. Plajer et al. [8] presented an active contouring algorithm for lung tumor segmentation based on a mixed internal-external force and on a cluster function. Awad et al. [9] used sparse field active models to segment 3-D lung tumors from CT image. Multi-parameter level set with a sphere shape prior was integrated in the model.

Up to now, lung tumor segmentation still remains as a notoriously challenging task considering the ability of a technique to segment the challenging types of tumors, the automation level of the technique and its robustness [10]. The main objective of this paper is to enhance automation and robustness of lung 4D-CT tumor segmentation, which is achieved by constructing a global graph of 4D-CT data and integrating context information constraint into the phases.

The graph cut algorithm has become one of the leading techniques among interactive methods since it has been proposed [11, 12]. The basic idea of the algorithm is to formulate the segmentation problem as an energy minimization problem [13, 14], which can be solved by max-flow/min-cut [15, 16]. The graph cut algorithm has been widely applied to the segmentation of bones, organs, vessels, and tumors, among others, in different medical modality images because it has many advantages [17–21]. The graph cut algorithm not only provides a globally optimal solution but can also easily incorporate various regional and boundary constraints.

A variety of studies have incorporated extra constraint information with the graph cut algorithm, such as shape priors, to achieve more accurate and robust segmentation results. Shape priors reduce the ambiguity by ruling out inconsistent segments with them and lead to improved results [22–25]. However, they only come into effect when the object fits a certain shape, which restricts their wide applications. Shape priors are not suitable for tumor segmentation. The irregular growth of tumor makes its size, shape, and location highly variable. Moreover, tumors typically show invasive growth, which likely includes peritumoral edema, and are prone to adhere to nearby tissues. Hence, tumor segmentation needs more specific and effective extra constraint information to guarantee accuracy.

**Fig 1:**
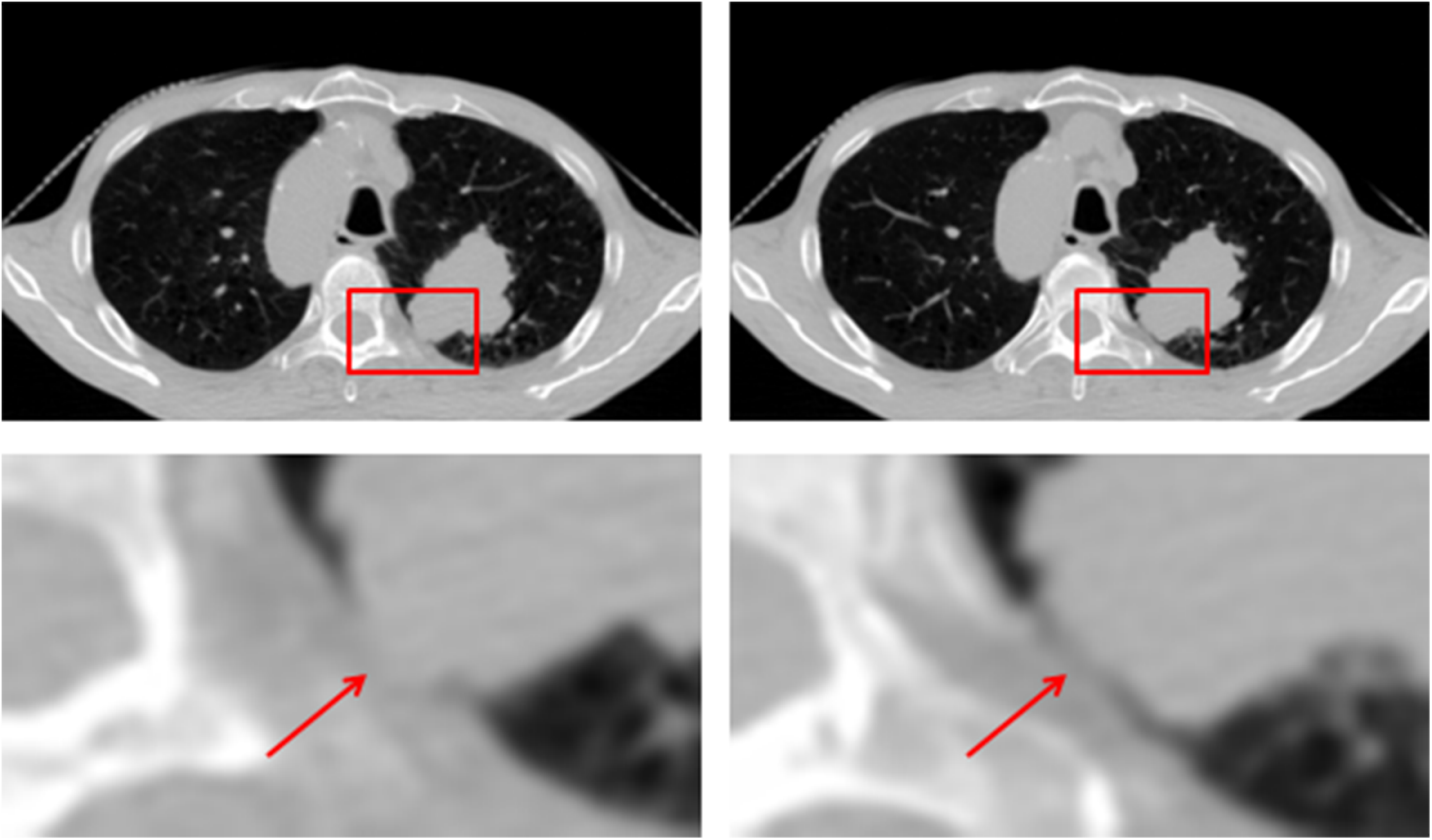
Weak edges in one phase become clear in the other phase. The same slices in different phases are shown in the first row. The areas in red boxes are enlarged and shown in the second row. The red arrows point to the tumor edges.

In a clinical setting, doctors always use the context information of lung 4D-CT when they manually contour the tumor. They focus not only on the image of the current phase but also on that of the adjacent phases. The context information helps doctors provide a more accurate target definition, especially when the tumor boundary of the current phase is weak and unclear or even missing. The image information lost in one respiratory phase may appear in other phases. Fig 1 shows an example of this situation.

Inspired by this idea, in this paper, we propose a novel segmentation method based on the graph cut framework for the lung 4D-CT tumors. We construct a global energy function, which contains regional terms, boundary terms of all phases, and new energy terms which make use of context information. Our global approach for the 4D-CT data provides two advantages. First, users only need to select object and background seeds in the slices of one phase and then the tumors in all phases can be simultaneously segmented. This approach decreases the interaction of users and relieves the doctors’ burden during radiotherapy. Second, a more accurate and robust segmentation can be achieved using context constraints. The results show that our method demonstrates better qualitative and quantitative performance than the graph cut algorithm without context constrains, the level set method [26] and the graph cut with star shape prior [24] compared with the ground truth provided by clinical experts.

The rest of our paper is organized as follows: the materials and the details of our method for the tumor segmentation of the lung 4D-CT data will be presented in Section II. The experimental results and evaluation are demonstrated in Section III. The discussion and conclusion are provided in Section IV and Section V.

## II. Materials and methods

### II.A. Lung 4D-CT data

This study was approved by the Ethics Committees of Nanfang Hospital, Southern Medical University. Patient records/information were anonymized and de-identified prior to analysis. We validate the proposed approach on ten different 4D-CT cases. The first two cases are from the DIR Lab at the University of Texas MD Anderson Cancer Center [27], which are acquired as part of the standard planning process for the treatment of thoracic malignancies by employing a GE medical system (General Electric Discovery DT PET/CT scanner). Each case contains ten phases, with a section spacing 2.5 mm, and a reconstruction matrix of 512 × 512 pixels. In-plane pixel spatial resolution is 0.97 × 0.97 mm. The third case comes from the Léon Bérard Cancer Center (Lyon, France) on a Philips 16-slice Brilliance Big Bore Oncology Configuration (Phillips Medical Systems, Cleveland, OH) [28]. It contains ten phases and the image size is 512 × 512 × 139 with a voxel size of 1.17 × 1.17 × 2.00 mm. The rest of cases are from the Nanfang Hospital of Southern Medical University. They are acquired by employing the Philips Brilliance CT Big Bore containing six phases. The in-plane grid size is 512 × 512, and the in-plane voxel dimension ranges from 0.83 × 0.83 mm to 1.10 × 1.10 mm. Table 1. shows the detailed information regarding the dataset.

**Table 1:** Summary of the 10 cases of the data that we consider.

Data Set	In-plane Grid Size	In-plane Dimensions (mm)	No. of Slices	Phase Number	Slice Spacing (mm)	Tumor Type
Texas 1	512×512	0.97 × 0.97	128	10	2.50	No tumor adhesion
Texas 2	512×512	0.97 × 0.97	120	10	2.50	Messy with adhesion
Lyon	512×512	1.17 × 1.17	139	10	2.00	Messy with adhesion
Nanfang 1	512×512	0.94 × 0.94	130	6	3.00	Messy with adhesion
Nanfang 2	512×512	0.86 × 0.86	144	6	3.00	Messy with adhesion
Nanfang 3	512×512	1.05 × 1.05	150	6	3.00	Messy with adhesion
Nanfang 4	512×512	0.94 × 0.94	125	6	3.00	Messy with adhesion
Nanfang 5	512×512	0.94 × 0.94	136	6	3.00	Messy with adhesion
Nanfang 6	512×512	1.10 × 1.10	138	6	3.00	Messy with adhesion
Nanfang 7	512×512	0.83 × 0.83	121	6	3.00	Messy with adhesion

### II.B. Graph cut method

A brief review of the graph cut method [11] is presented in this section.

Segmentation is achieved in this framework by first constructing a graph *G* = (*V*, *E*) corresponding to the image. *V* is a set of vertices representing the image voxels. Two additional terminal vertices exist in *V*, namely, *s* (source) and *t* (sink), which correspond to the object (tumor) and the background, respectively. *E* is a set of weighted edges representing the relationships of voxels in the image. Two types of edge sets are used, namely, t-links, the edges connecting the vertices to the terminals, and n-links, the edges connecting the neighboring vertices. The six-connected neighborhood system (*N*) is used here. Each edge *e*∈*E* has a non-negative weight *w*_*e*_ representing the penalty. The weights of the t-links represent the likelihood of a voxel belonging to the object or background region. They are obtained using the image intensity histogram modeled from the seeds provided by the user. The weights of the n-links represent the similarity between two neighboring voxels. The greater the similarity between the two neighboring voxels, the greater the weight of the edge connecting them. An *s*-*t* cut *C* is a subset of *E*. *V* is partitioned into two sets *S* (*s*∈*S*) and *T* = *V*−*S* (*t*∈*T*) when *C* is removed from *G*. Therefore, the cut in a graph represents the segmentation of the object region from the background region in the image. The cost of cut *C* is the sum of its edge weights: $|C|=\sum_{e\in c}w_{e}$. The minimum cut is the cut with the smallest cost and the objective is to determine the minimum cut and corresponding segmentation. The max-flow/min-cut algorithm can then be used.

The image segmentation is formulated as a binary labeling problem in this framework. Each voxel in the image is assigned a label from the label set *L* = {0, 1}, where 0 and 1 represent the background and the object, respectively. Let *v* be the voxel in the image and (*v*, *w*) be the voxel pairs. Let *f*_*v*_ ∈ *L* be the label assigned to voxel *v* and *f* = {*f*_*v*_ | *v* ∈ *V*} be the collection of all the label assignments. The energy function used for the segmentation is as follows:

$$E(f)=\sum_{v\in V}R_{v}(f_{v})+\lambda \sum_{(v,w)\in N}B_{vw}(f_{v},f_{w})$$ (1)

The first term on the right of Eq (1) is called the regional term because it represents the regional constraints. The term measures how well the voxels fit into the object or background models. *R*_*v*_(*f*_*v*_) is the penalty for assigning label *f*_*v*_ to voxel *v* relating to the weight of t-links. The second term on the right of Eq (1) is called the boundary term because it represents the boundary constraints. A segmentation boundary occurs if two neighboring voxels are assigned with different labels. *B*_*vw*_(*f*_*v*_,*f*_*w*_) is the penalty for assigning labels *f*_*v*_ and *f*_*w*_ to the neighboring voxels relating to the weight of the n-links. Parameter *λ* ≥ 0 is the coefficient to balance the regional and boundary terms. Smaller values of *λ* mean that the regional constraints are more dominant in Eq (1). The minimum cut and corresponding segmentation is found by minimizing the energy function Eq (1) using max-flow/min-cut algorithm.

### II.C. Lung tumor segmentation incorporating context information

We now show how to make use of the context information to construct a global graph of the lung 4D-CT to implement the multi-phase simultaneous segmentation of tumors.

**Fig 2:**
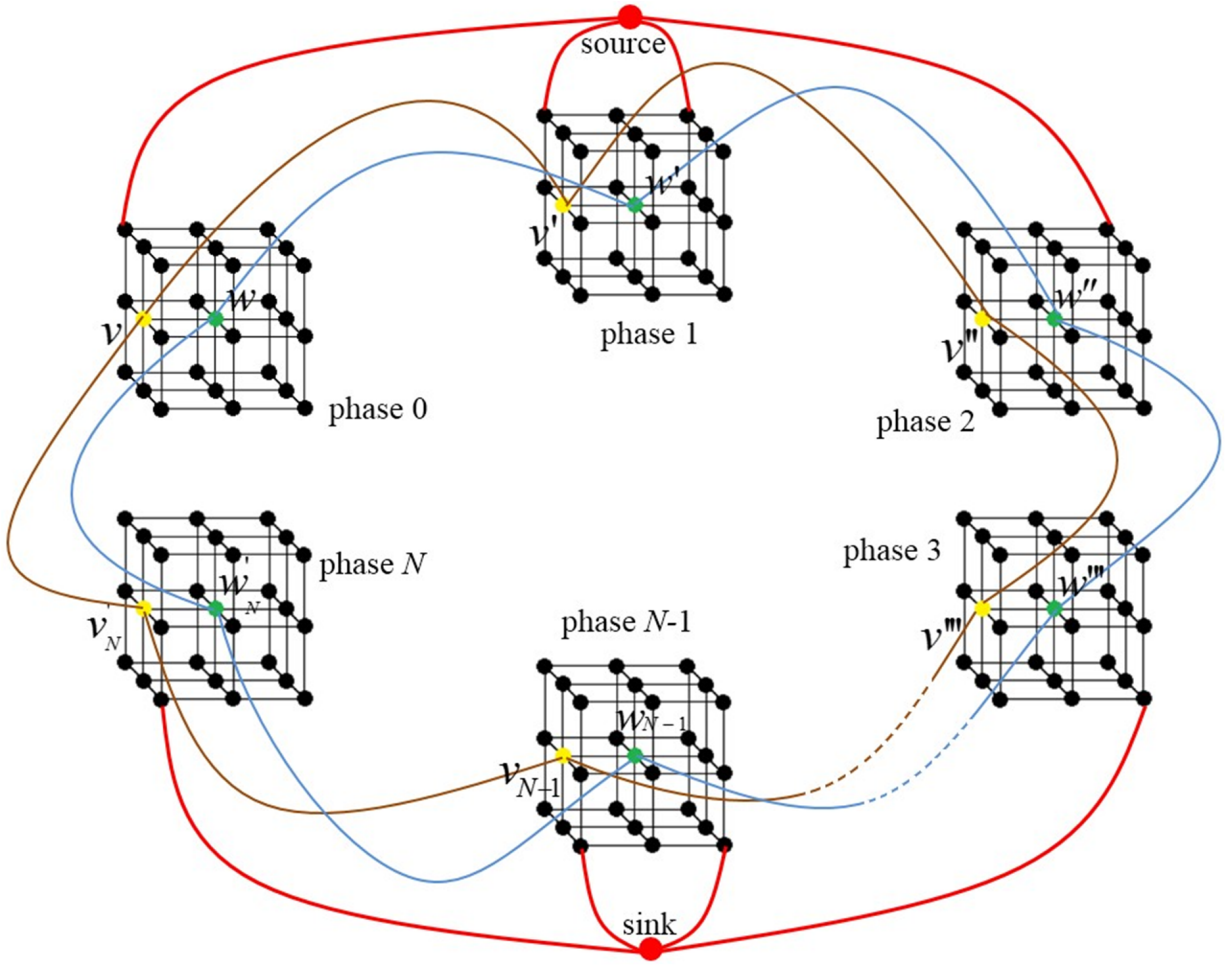
Global graph construction for simultaneous segmentation of lung 4D-CT. The black dots denote the voxels in the phase. The red dots denote the terminals: source and sink. The t- and n-links are indicated in red and black lines, respectively. Inter-subgraph arcs (blue and brown lines) denote the examples of added context information.

The proposed method formulates the tumor segmentation problem of the lung 4D-CT as a global graph based problem. The subgraphs for each phase are first constructed. The context information between the neighboring phases is then incorporated as a context constraint term, which is enforced by adding inter-subgraph arcs between the correspondent nodes of the neighboring subgraphs. Fig 2 shows the construction of global graph for lung 4D-CT.

The energy function for the single phase *I*_0_ is derived from the graph cut and comprises a regional term *R*_0,*v*_ and a boundary term *B*_0,(*v*,*w*)_. As commonly used, we set *R*_0,*v*_ as negative log-likelihoods:

$$R_{0,v}=\left\{ \begin{array}{l} -\ln\;\mathrm{Pr}(I_{0,v}|O),\;f_{v}=1\\ -\ln\;\mathrm{Pr}(I_{0,v}|B),\;f_{v}=0 \end{array}\right.$$ (2)

where *O* and *B* are the set of voxels marked by the user as object and background seeds, respectively. *f*_*v*_ is the assigned label for voxel *v*. *I*_0,*v*_ is the intensity of *v*. For any object seed *v* provided by the user, *R*_*v*_(0) = ∞, and for any background seed *v* provided by the user, *R*_*v*_(1) = ∞. Pr(*I*_0,*v*_ | *O*) and Pr(*I*_0,*v*_ | *B*) denote the probability of how well the unmarked voxel *v* fits into the histograms of the marked voxels for object and background intensity distribution, respectively. Notably, a similar intensity distribution of the tumor (object) is observed for each phase. Therefore, the *R*_0,*v*_ formulation can be used as a regional term of each other phase *I*_*i*_ denoted by *R*_*i*,*v*_.

We employ an ad-hoc function [11] with the following formulation for the cost design of the boundary term:

$$B_{0,(v,w)}=\lambda_{1}\exp(-\frac{{\left(I_{0,v}-I_{0,w}\right)}^{2}}{2\sigma_{1}{}^{2}})\cdot \frac{1}{dist(v,w)}$$ (3)

where *σ*_1_ is a given Gaussian parameter, and *λ*_1_ is the scaling constant. Similarly, the boundary cost *B*_*i*,(*v*,*w*)_ for each other phase would have the same formulation.

A context term is introduced to make the best incorporation of the context information of the lung 4D-CT data. We consider the case of the Potts model [13], i.e., $B_{0,(v,v^{'})}=C_{0,(v,v^{'})}\cdot T(f_{v}\neq f_{v^{'}})$ for single phase *I*_0_. Voxels *v* and *v′* are the corresponding voxels from *I*_0_ and its neighboring phase *I*_1_. We can incorporate contextual information into our energy function by allowing $C_{0,(v,v^{'})}$ to vary depending on intensities *I*_0,*v*_ and $I_{1,v^{'}}$. We then define

$$C_{0,(v,v^{'})}=\left\{ \begin{array}{l} 2\lambda_{2}\sigma_{2}\;\text{if}\;|I_{0,v}-I_{1,v^{'}}|\leq \tau \\ \lambda_{2}\sigma_{2}\;\text{if}\;|I_{0,v}-I_{1,v^{'}}|>\tau \end{array}\right.$$ (4)

where $C_{0,(v,v^{'})}$ is employed to penalize the disagreement $\left(f_{v}\neq f_{v^{'}}\right)$ between the labels of the corresponding voxels *v* and *v′*; *σ*_2_ is the Potts model parameter; *τ* is the threshold parameter; and *λ*_2_ is the scaling constant. Similarly, the contextual cost $C_{i,(v,v^{'})}$ for each other phase would have the same formulation.

We have so far defined the regional and boundary terms for each phase. The contextual term relative to the inter-phase is also defined. We can then achieve the global graph energy function for the lung 4D-CT tumor segmentation in the following method:

$$E_{g}(f)=\sum_{i=0}^{k}\left\{R_{i,v}(f_{v})+B_{i,(v,w)}(f_{v},f_{w})+C_{i,(v,v^{'})}(f_{v},f_{v^{'}})\right\}$$ (5)

where *k* is the number of phases in the lung 4D-CT. The optimal solution of this new energy function can be found by computing the max-flow/min-cut algorithm. In this study, we employed Boykov-Kolmogorov’s algorithm [15] to find the max-flow/min-cut, since this algorithm has been proven to be effective [16]. Eq (5) makes use of the context information of 4D-CT to complete multi-phase tumor simultaneous segmentation. The tumors in the images of each phase can be concurrently segmented with the object and background seeds selected by users in one phase.

### II. D. Evaluation strategy

Both the visual and quantitative results will be presented to demonstrate the performance of different methods. For quantitative evaluation, the segmentation performance is compared with the ground truth. The ground truth are the manual contours delineated by experienced radiation oncologists applying the Eclipse (Varian) or Monaco (Elekta) radiotherapy planning system. It usually takes doctors approximately 30 minutes to accomplish target delineation of one lung 4D-CT dataset.

The segmentation result is termed *A*, whereas the ground truth is termed *G*. We use the dice similarity coefficient (DSC) for the volumetric error measurement and the average symmetric surface distance (ASSD) for the boundary surface distance error. All DSC and ASSD values are computed in 3D [29].

The DSC values are obtained using:

$$\text{DSC}=\frac{2|A\cap G|}{\left|A|+|G\right|}$$ (6)

The DSC values range between 0 and 1. DSC = 0 means the segmentation result and the ground truth has no overlap at all, whereas 1 indicates their perfect agreement.

The ASSD values are obtained using:

$$\text{ASSD}=\frac{\sum_{a\in A}\min_{b\in G}dist(a,b)+\sum_{b\in G}\min_{a\in A}dist(a,b)}{N_{A}+N_{G}}$$ (7)

where *a* and *b* are the border voxels on the segmentation result surface and the ground truth surface, respectively. *dist*(*a*, *b*) represents the distance between *a* and *b*. *N*_*A*_ and *N*_*G*_ are the number of border voxels on *A* and *G*, respectively. A small ASSD value indicates an accurate segmentation. ASSD = 0 means a perfect segmentation.

## III. Results

### III.A. Initialization

**Fig 3:**
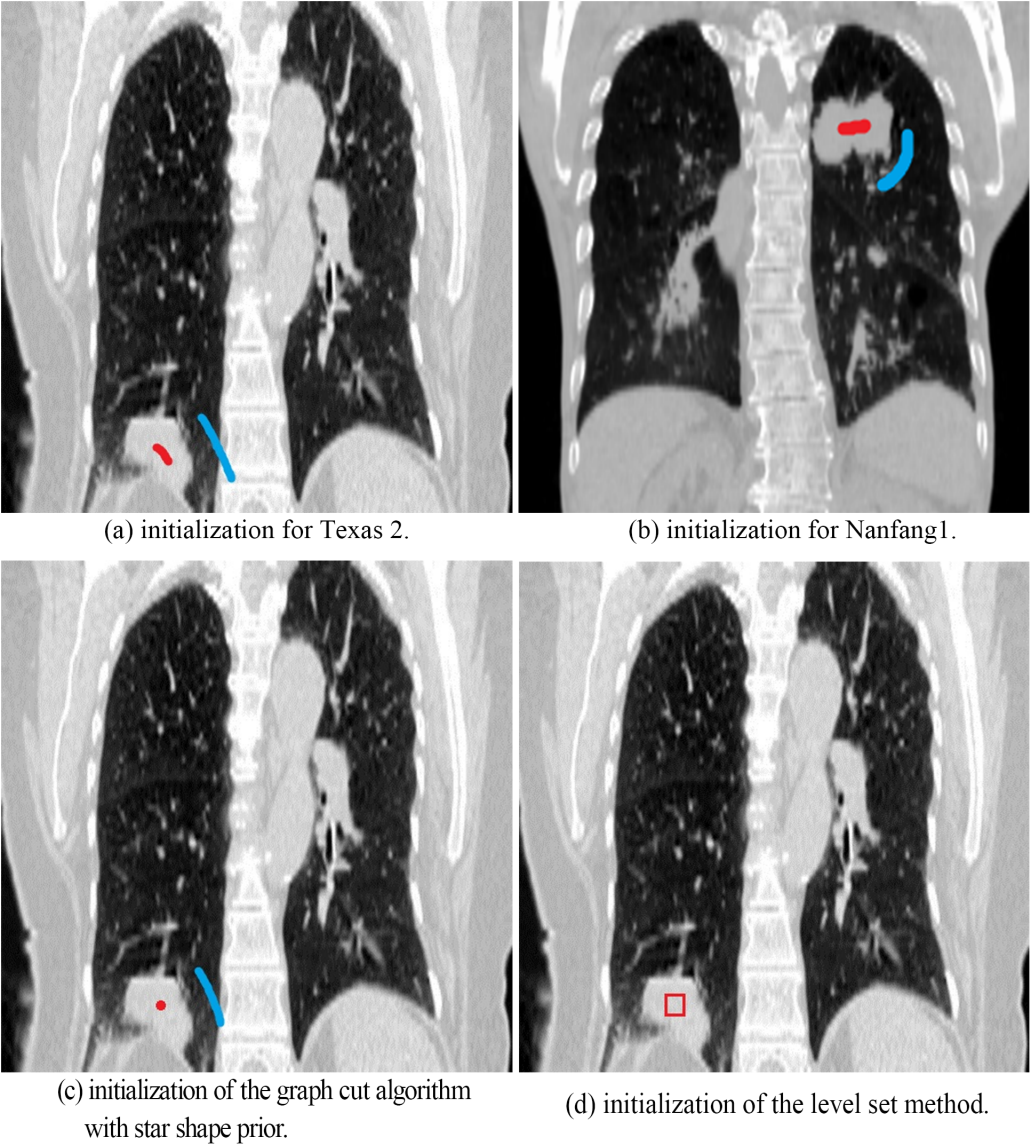
Example of initializations. Red: object seeds. Blue: background seeds.

The initialization of our method is accomplished by asking the doctor to identify the tumor location and select object and background seeds only in one respiratory phase (Fig 3(A) and 3(B)). This is the only step requiring user interaction. Segmentation is performed afterward, and the results of each phase are generated. The manual contours by the experienced oncologist are used to demonstrate the performance of our method in comparison to the graph cut without context constrain, the level set method, and the graph cut with star shape prior. The initialization of graph cut algorithm with star shape prior is similar to our proposed method (Fig 3(C)). However, it is necessary to select the background and foreground points at each phase. The initialization contour of the level set method [26] is a rectangle, as shown in Fig 3(D).

### III.B. Visual inspection

**Fig 4:**
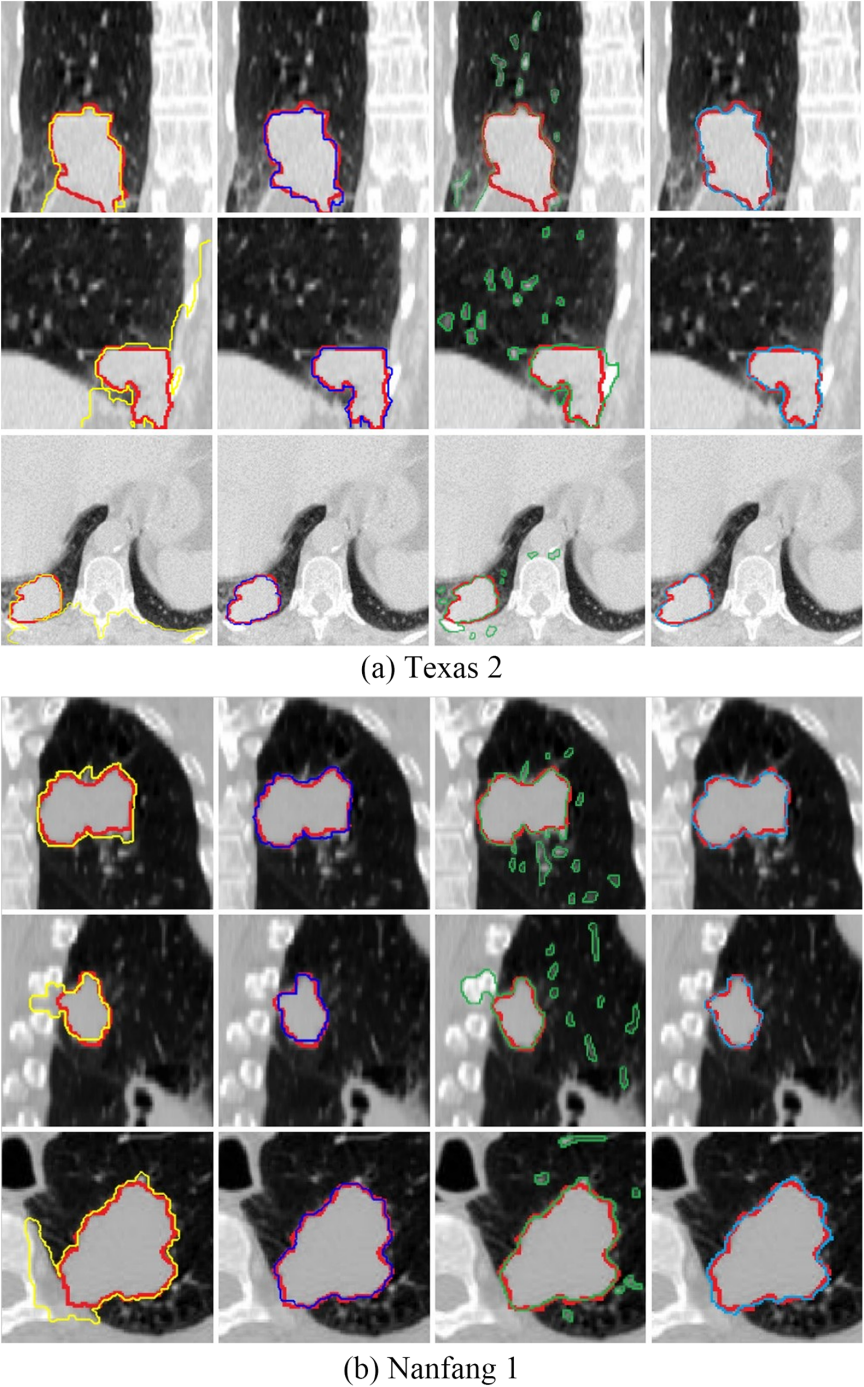
Visual comparison of segmentation results given by different methods. The 1st column: the graph cut without context constrains (yellow) and ground truth (red) are overlaid. The 2nd column: our proposed method (deep blue) and ground truth (red) are overlaid. The 3rd column: The level set method (green) and ground truth (red) are overlaid. The 4th column: the graph cut with star shape prior (pale blue) and ground truth (red) are overlaid.

Fig 4 shows the representative segmentation results. Different views, namely, coronal, sagittal, and transverse, are demonstrated from top to bottom. The ground truth is shown in red in all images. The segmentation results of the graph cut without context constrains (yellow), our proposed method (deep blue), the level set method (green) and the graph cut with star shape prior (pale blue) are shown in the first, second, third and fourth columns, respectively.

From Fig 4, we can see that when the level set method is applied, leaking problems can be clearly observed. For the graph cut without context constrains method, the unsatisfying performance is most likely caused by the weak boundary due to the low intensity contrast between tumor and its surroundings. In comparison with the level set method and the graph cut without context constrains, the proposed approach works well. Using the context information of lung 4D-CT, our proposed method provides more accurate and robust segmentation results, exhibiting expected improvement. In comparison with our proposed method, the graph cut with star shape prior provides similar segmentation results in visualization. However, tumors in each phase are segmented simultaneously by our method with little user interaction due to the construction of the global graph, which is what the single graph cannot accomplish.

### III. C. Quantitative evaluation

**Fig 5:**
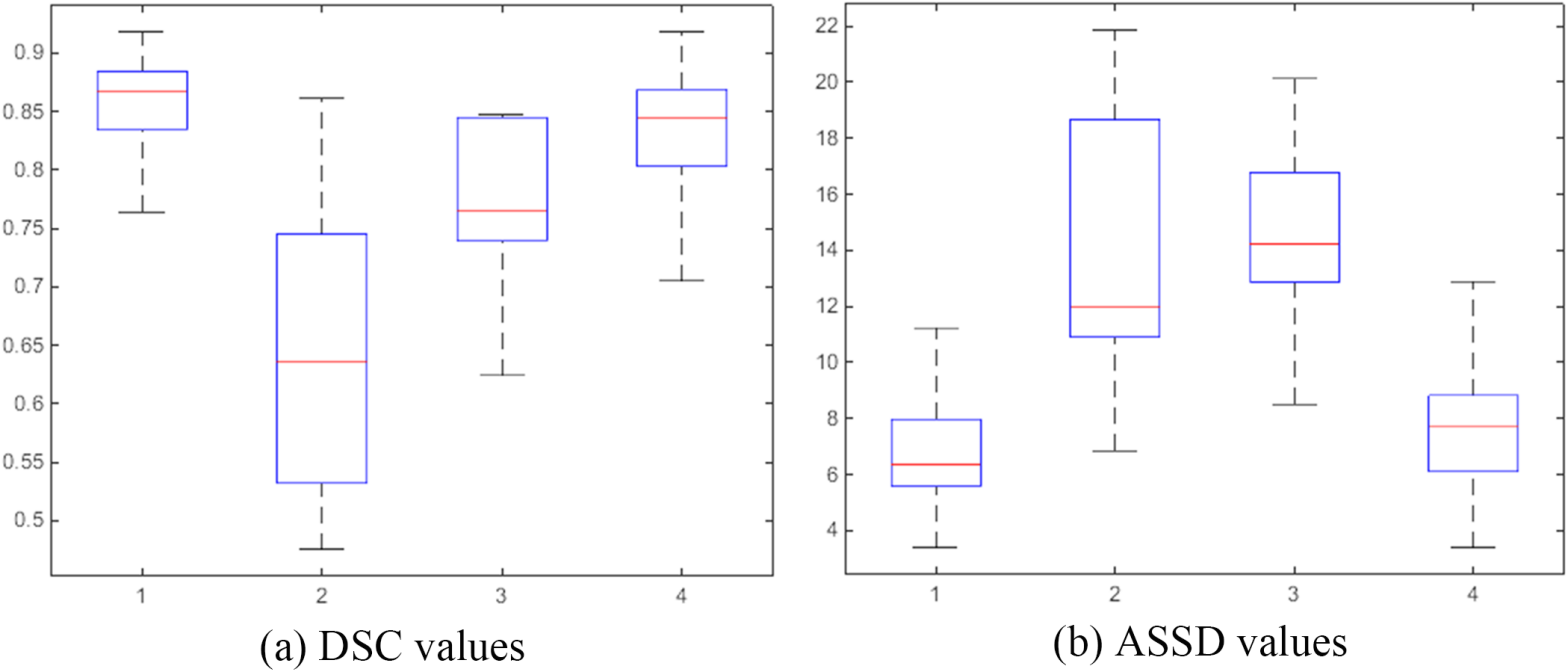
Quantitative and comparative performance evaluation. 1: our proposed method, 2: the graph cut without context constrains, 3: the level set method, 4: the graph cut with star shape prior.

Fig 5 shows the quantitative comparison for all 10 lung 4D-CT dataset among the four methods. It is clear that in these cases our method achieves the highest DSC and lowest ASSD values. Our method obtained tumor segmentation accuracy characterized by DSC = 0.855±0.048 on average, which was higher than that of the graph cut without context constraints (0.638±0.119), the level set method (0.763±0.072) and the graph cut with star shape prior (0.831±0.068). The average ASSD value of our method was 6.88±2.35, which was lower than that of the graph cut without context constraints (13.73±4.83), the level set method (14.49±2.99) and the graph cut with star shape prior (7.80±2.86).

The proposed method utilizing the context information of 4D-CT outperformed the other three methods and provided more accurate and robust segmentation results. The performances exhibited expected improvement and indicated that our method has the ability to more accurately segment the challenging types of tumors.

## IV. Discussion

We presented a lung 4D-CT tumor data segmentation algorithm in this paper. We utilized the characters of the lung 4D-CT data to construct a global energy function, which combined the sub-graph of each phase and the contextual cost terms between the neighboring phases. We reduced the user interaction and increased the accuracy and robustness of tumor segmentation. Our method was tested on different 4D-CT cases and demonstrated consistent improvements over the graph cut without context constrains, the level set method and the graph cut with star shape prior in visual evaluation and quantitative assessment.

### IV.A. Parameter setting

**Fig 6:**
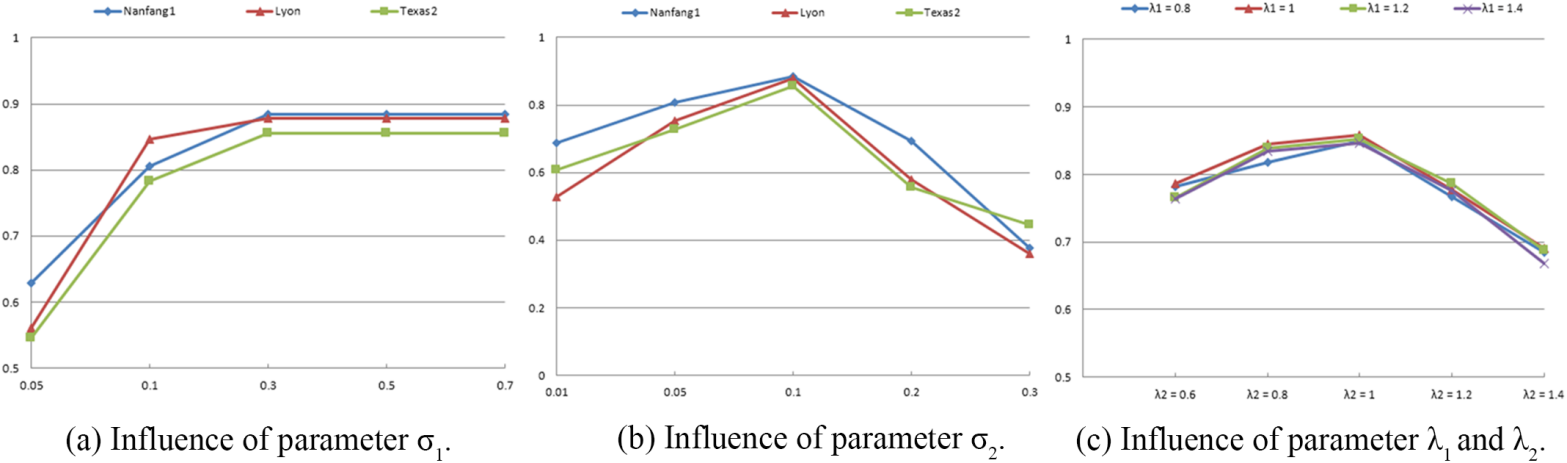
Influence of parameters.

To achieve the best segmentation for our proposed approach, several critical parameters need to be fine-tuned. We used several cases to test the influence of parameter *λ*_1_, *λ*_2_, *σ*_1_ and *σ*_2_. The DSC values with respect to the use of different parameter values are shown in Fig 6(A), 6(B) and 6(C). It is clear that *σ*_1_ = 0.5, *σ*_2_ = 0.1 and *λ*_2_ = 1 provided the better segmentation results in term of DSC values. Meanwhile, it can be seen that the segmentation results seemed not to be strongly affected by the setting of *λ*_1_. Hence, we set *λ*_1_ = *λ*_2_ = 1, *σ*_1_ = 0.5 and *σ*_2_ = 0.1 throughout all experiments. The value of parameter *τ* was set according to the default parameter value given in [13].

### IV.B. Robustness to initialization

**Fig 7:**
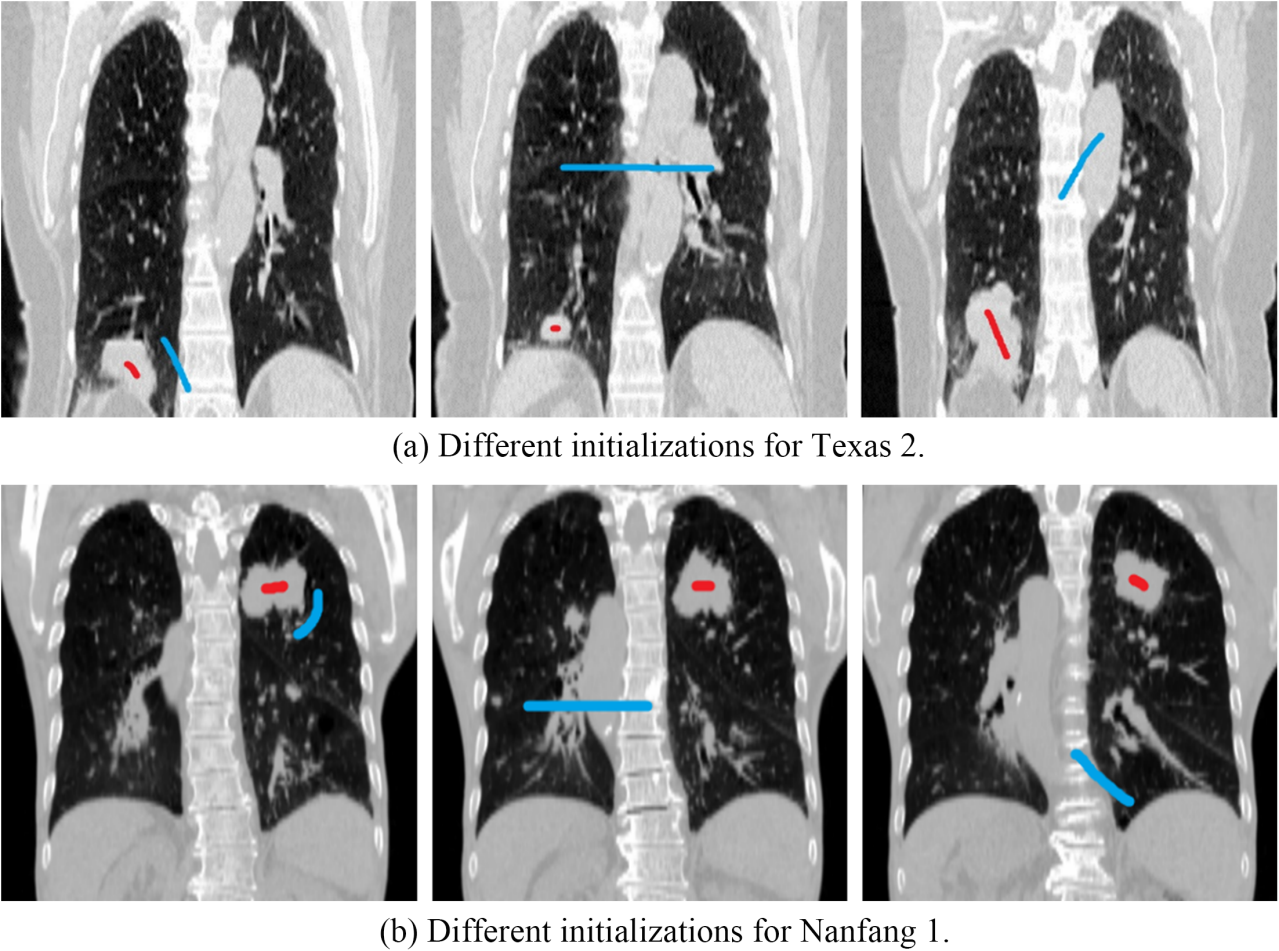
Examples of different initializations. Red: object seeds. Blue: background seeds.

**Fig 8:**
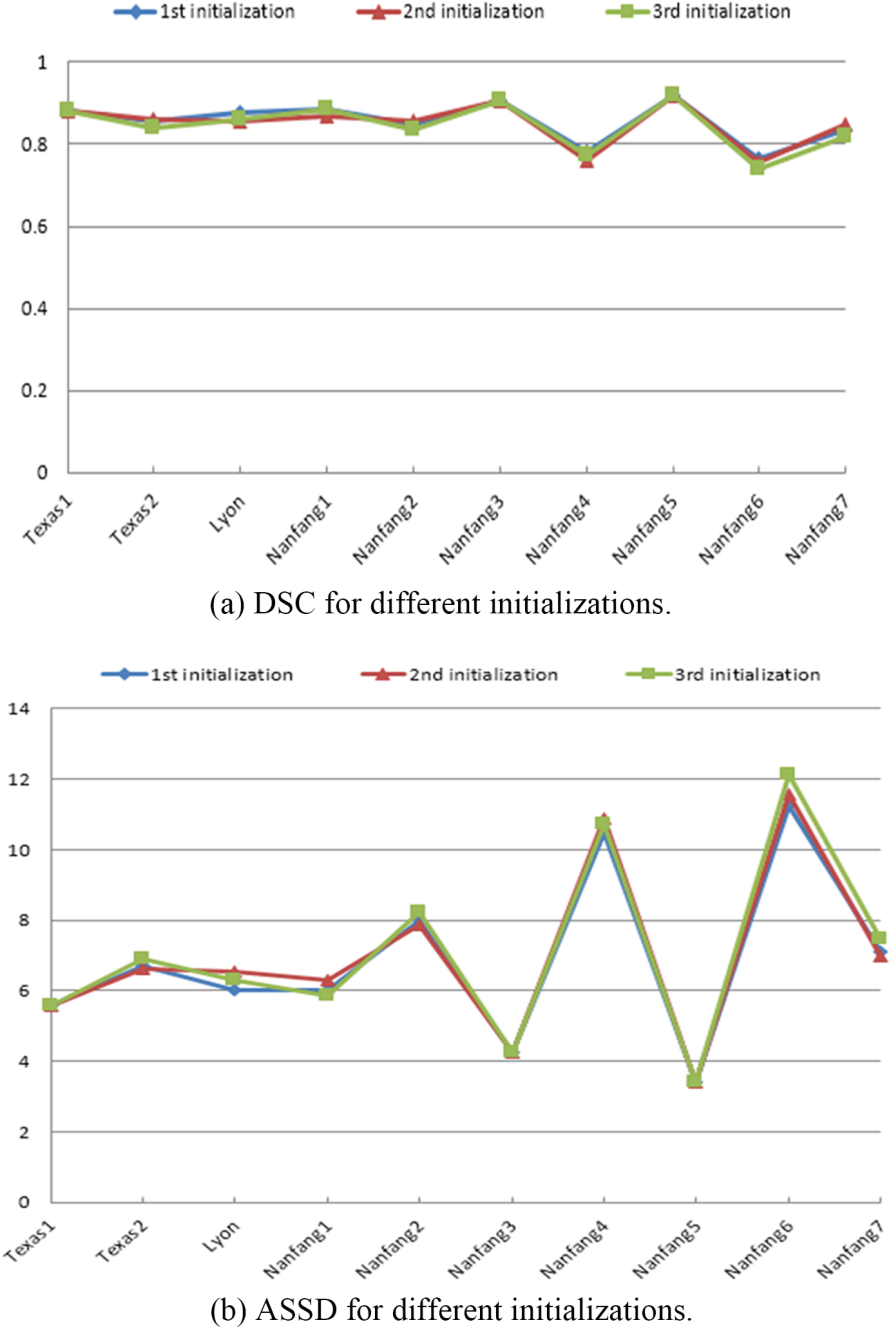
Quantitative results for our method using different initializations.

The proposed algorithm was implemented in Matlab with a graphical user interface (GUI). The sole user interaction involved was selecting object and background seeds on a slice in one phase using a 2D brush tool. Different users were asked to perform the initializations independently on the whole datasets. We tested the segmentation accuracy varies relative to different initializations on the whole datasets. Fig 7 shows the examples of the three different initial selections of two cases. Fig 8 shows the DSC and ASSD of the segmented results with respect to the three different initializations. It can be seen that our proposed method achieved highly stable results over those three initializations.

### IV. C. Future work

Our algorithm was implemented in Matlab language on a standard PC with a 3.20 GHz Intel Core i5-6500 CPU and 8 GB memory, running 64-bit Windows system. Current implementation required about 30 minutes for each dataset. We’re aware that the current code is not fully optimized, it could be improved further in the future work.

The initialization of the object and background seeds can actually be automated. Threshold and clustering are usually proposed for automatic initialization, which further alleviates the human burden in specifying the object and background seeds [30, 31].

Lung tumors usually adhere to nearby vessels, diaphragm, and liver, among others, because of their invasive growth. Leaking problems are prone to happen when segmenting such a tumor. However, the boundary surface of structures around the tumor can serve as valuable prior information to help segmentation. Hence, we plan to obtain those boundary surfaces in the future and make use of them to improve the segmentation results.

Moreover, we tend to apply machine-learning methods based on training datasets in the future to find appropriate parameter settings although those parameters are empirically determined in this study.

In this study, we focused only on the lung 4D-CT tumor segmentation. It also may have potential applications to other 4D medical images (e.g., 4D MRI and 4D ultrasound). Our future study will focus on further improving the proposed method and evaluating the feasibility of applying our method to multimodality images.

## V. Conclusion

Computer-aided segmentation techniques can greatly benefit target definition in cancer radiation therapy. The proposed method addressed this problem by providing a graph cut-based approach, which utilized the context information of the lung 4D-CT. Compared with previous studies, our method offered an effective method of obtaining more accurate and robust segmentation results. Our method can be especially useful for lung tumors characterized by weak edges and low contrast with surrounding normal structures. As long as doctors select object and background seeds in the image of one phase rather than in each phase, tumors in each phase will be simultaneously segmented, which has the potential to greatly decrease the time for clinicians to perform target definition.

## References

[1] Keall P, Mageras G, Balter J, Emery R, Forster K, Jiang S, et al The management of respiratory motion in radiation oncology report of AAPM Task Group 76. Medical Physics. 2006; 33(10): 3874–3900. 17089851 10.1118/1.2349696

[2] Li R, Lewis J, Cervino L, Jiang S. 4D-CT sorting based on patient internal anatomy. Physics in Medicine and Biology. 2009; 54(15): 4821–4833. doi: 10.1088/0031-9155/54/15/012 19622855 10.1088/0031-9155/54/15/012

[3] Georg M, Souvenir R, Hope A, Pless R. Manifold learning for 4D CT reconstruction of the lung. IEEE Computer Society Conference on Computer Vision and Pattern Recognition Workshops. 2008: 1–8.

[4] Rietzel E, Chen G, Choi N, Willet C. Four-dimensional image-based treatment planning: target volume segmentation and dose calculation in the presence of respiratory motion. International Journal of Radiation Oncology Biology Physics. 2005; 61(5):1535–1550. 10.1016/j.ijrobp.2004.11.037 15817360

[5] Cui H, Wang X, Fulham M, Feng D. Prior knowledge enhanced random walk for lung tumor segmentation from low-contrast CT images. IEEE Engineering in Medicine and Biology Society. 2013: 6071–6074. 10.1109/EMBC.2013.6610937 24111124

[6] Gu Y, Kumar V, Hall L, Goldgof D, Li C, Korn R, et al Automated delineation of lung tumors from CT images using a single click ensemble segmentation approach. Pattern Recognition. 2013; 46(3): 692–702. doi: 10.1016/j.patcog.2012.10.005 23459617 10.1016/j.patcog.2012.10.005 PMC3580869

[7] Vivanti R, Joskowicz L, Karaaslan O, Sosna J. Automatic lung tumor segmentation with leaks removal in follow-up ct studies. International Journal of Computer Assisted Radiology and Surgery. 2015; 10(9): 1505–1514. doi: 10.1007/s11548-015-1150-0 25605297 10.1007/s11548-015-1150-0

[8] Plajer I, Richter D. A new approach to model based active contours in lung tumor segmentation in 3D-CT image data. IEEE International Conference on Information Technology and Applications in Biomedicine. 2010:1–4.

[9] Awad J, Wilson L, Parraga G, Fenster A. Lung tumors segmentation on CT using sparse field active model. Proceedings of SPIE—The International Society for Optical Engineering, 2011; 7963(3): 345–353.

[10] El-Baz A, Beache G, Gimel'Farb G, Suzuki K, Okada K, Elnakib A, et al Computer-aided diagnosis systems for lung cancer: challenges and methodologies. International Journal of Biomedical Imaging. 2013; (1): 942353–942353. doi: 10.1155/2013/942353 23431282 10.1155/2013/942353 PMC3570946

[11] Boykov Y, Jolly M. Interactive Graph Cuts for Optimal Boundary & Region Segmentation of Objects in N-D Images. IEEE International Conference on Computer Vision. 2001; (1): 105–112.

[12] Boykov Y, Jolly M. Interactive Organ Segmentation Using Graph Cuts. Medical Image Computing and Computer Assisted Intervention. 2000; (1935): 276–286.

[13] Boykov Y, Veksler O, Zabih R. Fast approximate energy minimization via graph cuts. IEEE Transactions on Pattern Analysis & Machine Intelligence. 2001; 23(11): 1222–1239.

[14] Kolmogorov V, Zabih R. What Energy Functions Can Be Minimized via Graph Cuts?. European Conference on Computer Vision 2002; (26): 65–81. 10.1109/TPAMI.2004.1262177 15376891

[15] Boykov Y, Kolmogorov V. An experimental comparison of min-cut/max-flow algorithms for energy minimization in vision. IEEE Transactions on Pattern Analysis & Machine Intelligence. 2004; 26(9): 1124–1137. 15742889 10.1109/TPAMI.2004.60

[16] Boykov Y, Funka-Lea G. Graph cuts and efficient N-D image segmentation. International Journal of Computer Vision. 2006; 70(2): 109–131.

[17] Ju W, Xiang D, Zhang B, Wang L, Kopriva I, Chen X. Random walk and graph cut for co-segmentation of lung tumor on PET-CT images. IEEE Transactions on Image Processing. 2015; 24(12): 5854–5867. doi: 10.1109/TIP.2015.2488902 26462198 10.1109/TIP.2015.2488902

[18] Guo F, Jing Y, Rajchl M, Svenningsen S, Picapaldi D, Sheikh K, et al (2015). Globally optimal co-segmentation of three-dimensional pulmonary ^1^h and hyperpolarized ^3^he MRI with spatial consistence prior. Medical Image Analysis. 2015; 23(1): 43–55. doi: 10.1016/j.media.2015.04.001 25958028 10.1016/j.media.2015.04.001

[19] Ballangan C, Wang X, Fulham M, Eberl S, Feng D. Lung tumor segmentation in PET images using graph cuts. Computer Methods and Programs in Biomedicine. 2013; 109(3): 260–268. doi: 10.1016/j.cmpb.2012.10.009 23146420 10.1016/j.cmpb.2012.10.009

[20] Han D, Bayouth J, Song Q, Taurani A, Sonka M, Buatti J, et al Globally optimal tumor segmentation in PET-CT images: a graph-based co-segmentation method. International Conference on Information Processing in Medical Imaging. 2011; (22): 245–256. 10.1007/978-3-642-22092-0_21 PMC3158679 21761661

[21] Qi S, Junjie B, Dongfeng H, Sudershan B, Wenqing S, William R, et al Optimal co-segmentation of tumor in PET-CT images with context information. IEEE Transactions on Medical Imaging. 2013; 32(9): 1685–1697. doi: 10.1109/TMI.2013.2263388 23693127 10.1109/TMI.2013.2263388 PMC3965345

[22] Das P, Veksler O, Zavadsky V, Boykov Y. Semiautomatic segmentation with compact shape prior. Image and Vision Computing. 2009; 27(1): 206–219.

[23] Malcolm J, Rathi Y, Tannenbaum A. Graph Cut Segmentation with Nonlinear Shape Priors. IEEE International Conference on Image Processing. 2007; (4): 365–368.

[24] Veksler O. Star Shape Prior for Graph-Cut Image Segmentation. European Conference on Computer Vision. 2008; (5304):454–467.

[25] Wang H, Zhang H, Ray N. Adaptive shape prior in graph cut image segmentation. Pattern Recognition. 2013; 46(5): 1409–1414.

[26] Li C, Huang R, Ding Z, Gatenby J, Metaxas D, Gore J. A level set method for image segmentation in the presence of intensity inhomogeneities with application to MRI. IEEE Transactions on Image Processing. 2011; 20(7): 2007–2016. doi: 10.1109/TIP.2011.2146190 21518662 10.1109/TIP.2011.2146190 PMC6952214

[27] Castillo R, Castillo E, Johnson V, Mcphail T, Garg A, Guerrero T. A framework for evaluation of deformable image registration spatial accuracy using large landmark point sets. Physics in Medicine and Biology. 2009; 54(7): 1849–1870. doi: 10.1088/0031-9155/54/7/001 19265208 10.1088/0031-9155/54/7/001

[28] Vandemeulebroucke J, Rit S, Kybic J, Clarysse P, Sarrut D. Spatiotemporal motion estimation for respiratory correlated imaging of the lungs. Medical Physics. 2011; 38(1): 166–178. doi: 10.1118/1.3523619 21361185 10.1118/1.3523619

[29] Ginneken B, Heimann T, Styner M. 3D segmentation in the clinic: a grand challenge. Medical Image Computing and Computer Assisted Intervention. 2007; 39(4): 93–95.

[30] Montgomery D, Amira A. Fully automated segmentation of oncological PET volumes using a combined multiscale and statistical model. Medical Physics. 2007; 34(2): 722–736. doi: 10.1118/1.2432404 17388190 10.1118/1.2432404

[31] Bagci U, Udupa J, Mendhiratta N, Foster B, Xu Z, Yao J, et al Joint segmentation of anatomical and functional images: applications in quantification of lesions from PET, PET-CT, MRI-PET, and MRI-PET-CT images. Medical Image Analysis. 2013; 17(8): 929–945. doi: 10.1016/j.media.2013.05.004 23837967 10.1016/j.media.2013.05.004 PMC3795997

